# Effects of 1-week inpatient multidisciplinary care for chronic kidney disease prior to outpatient collaborative care

**DOI:** 10.1007/s10157-024-02496-5

**Published:** 2024-04-20

**Authors:** Natsuko Okuno, Hiroshi Kado, Hiroyoshi Segawa, Tsuguru Hatta

**Affiliations:** 1 Department of Nephrology, Omihachiman Community Medical Center, 1379 Tsuchida-Cho, Omihachiman, Shiga Japan; 2 Department of Nephrology, Kyoto Prefectural University of Medicine, 465 Kajii-Cho, Kamigyo-Ku, Kyoto Japan

**Keywords:** Unplanned induction, Hemodialysis using a central venous catheter, Postponing dialysis induction

## Abstract

**Background:**

Multidisciplinary care for Chronic Kidney Disease (CKD) has been reported to be effective in preventing deterioration of renal function and avoiding hemodialysis induction using a central venous catheter.

**Methods:**

We included 171 patients who received dialysis at our department between October 2014 and June 2017. Patients were divided into two groups: an inpatient group who received inpatient multidisciplinary care for CKD (educational hospitalization) prior to outpatient collaborative care from their family physician and nephrologist, and a non-inpatient group who did not receive such care. We compared factors related to dialysis induction.

**Results:**

There was no significant difference in eGFR between the groups at the start of observation. The mean time from the start of observation to dialysis induction (inpatient group vs. non-inpatient group; 40.8 ± 2.8 vs. 23.9 ± 3.0 months, respectively; *P* < 0.001) and the rate of hemodialysis induction using a central venous catheter (22.5 vs. 47.1%, respectively; *P* = 0.002) were significantly different between the groups. Survival analysis showed that the time to dialysis induction was significantly longer in the inpatient group (*P* = 0.0001). Multivariate analysis revealed that educational hospitalization (odds ratio = 0.30 [95% CI 0.13, 0.67]) was significantly associated with hemodialysis induction using a central venous catheter.

**Conclusion:**

Educational hospitalization prior to outpatient collaborative care is beneficial for preventing hemodialysis induction using a central venous catheter and postponing dialysis induction.

**Supplementary Information:**

The online version contains supplementary material available at 10.1007/s10157-024-02496-5.

## Introduction

Chronic kidney disease (CKD) is a disease for which continuous multidisciplinary care, including nephrologists, nurses, pharmacists, and dietitians, is important. Multidisciplinary care for CKD has been reported to prevent deterioration of renal function and avoid the induction of hemodialysis using a central venous catheter, factors that worsen prognosis during dialysis [1–8]. Multidisciplinary care can be categorized into outpatient and inpatient multidisciplinary care. Most previous studies have reported the effectiveness of outpatient multidisciplinary care; however, only two studies reported the effectiveness of inpatient multidisciplinary care [2, 6, 7].

When initiating interventions for CKD patients, it is advantageous to begin with intensive, multidisciplinary care in an inpatient setting. This approach helps to motivate patients and enhances their understanding of the disease, which can then be leveraged for subsequent outpatient interventions. At our hospital, most CKD patients who are referred to the Nephrology department by their family physicians receive 1 week of inpatient multidisciplinary care as soon as possible after the referral, which is called “Educational Hospitalization” (EH). All CKD patients who are referred to our department are recommended to receive EH, and it is strongly recommended for patients with progressively worsening renal function and those with inadequate control of factors associated with deterioration of renal function such as urinary protein and blood pressure.

Acquisition of accurate disease knowledge for self-management are key themes of EH. To help patients understand CKD, a multidisciplinary team consisting of nephrologists, ward nurses, dialysis nurses, pharmacists, dietitians, physical therapists, and clinical engineers is organized, and lectures and guidance are provided to the team with the aim of improving knowledge of the disease and awareness of treatment during hospitalization. The schedule for EH is shown in Supplementary Table 1. After EH, the institute’s physicians (consisting of nephrologists and internal medicine physicians trained under nephrologists) and the patient’s family physicians collaborate to provide continued outpatient multidisciplinary care. During the period of collaborative care, the family physician continues to provide similar regular outpatient medical care as before the referral, and our institute continues to provide regular outpatient medical care every 3–6 months. Patients are instructed to maintain records of home blood pressure and urine storage tests, which are kept at their home, to assess blood pressure, proteinuria, and salt intake. Based on the results of these tests, ongoing management of blood pressure and proteinuria is continued. Outpatient nutritional guidance, focusing on salt reduction, is provided as needed at the physician’s discretion.

In this study, CKD patients who had started maintenance dialysis after attending our department were divided into two groups: those who received CKD intervention starting with EH (inpatient group), and those who received CKD intervention without EH (non-inpatient group). We examined the effect of EH on factors related to dialysis induction between the groups.

## Methods

### Study design and population

**Fig. 1:**
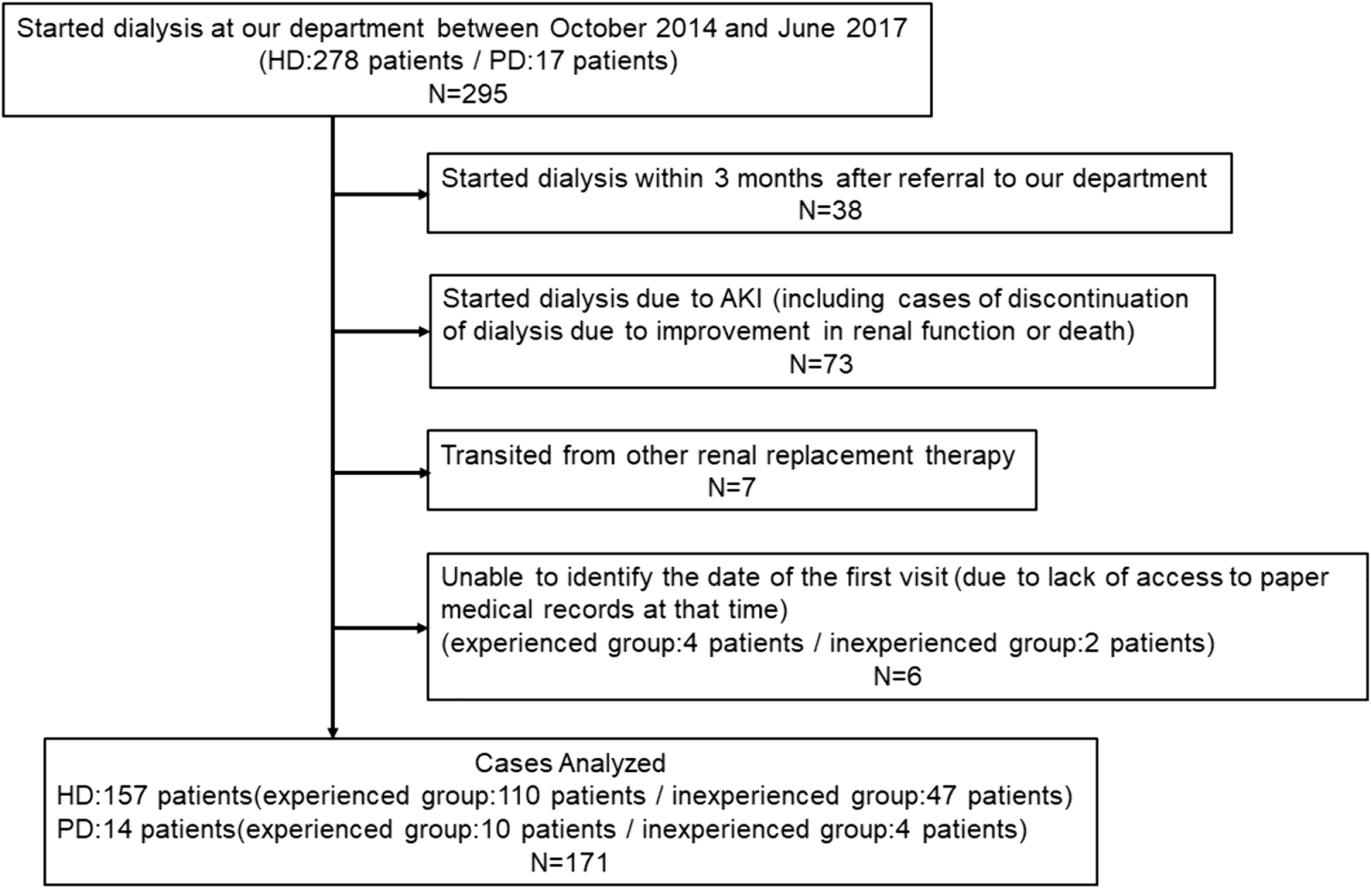
Flowchart of the analysis subject selection

A cohort study was conducted. A total of 295 patients who started dialysis at the Department of Nephrology of Omihachiman City Medical Center between October 2014 and June 2017 were enrolled. Exclusion criteria were: (1) patients who started dialysis within 3 months after referral to our department, (2) patients who started dialysis due to acute kidney injury, (3) patients who withdrew from dialysis or died before discharge, (4) patients who were transferred to hemodialysis from peritoneal dialysis or renal transplantation, and (5) patients who could not identify the date of their first visit because the corresponding paper medical records could not be referenced (our medical records were converted from paper to electronic records in 2006). The subject selection process is shown in Fig. 1.

A total of 171 patients consisting of 157 patients who started maintenance dialysis and 14 patients who started peritoneum dialysis were included in this study. The patients were divided into an inpatient group who received educational hospitalization early in the intervention, followed by collaborative care from our physicians and family physicians until dialysis induction; and a non-inpatient group who were followed up only by collaborative care from our physicians and family physicians from the first visit to dialysis induction.

Our physicians consisted of nephrologists and physicians who had been fully trained by nephrologists.

Both groups received multidisciplinary care during the outpatient collaborative care period. Interventions for each group during the observation period are shown in Supplementary Fig. 1.

### Clinical data collection

The following medical information and laboratory data were collected from electronic medical records: baseline characteristics; Estimated Glomerular Filtration Rate (eGFR) at the start of observation; eGFR at dialysis induction; time from the start of observation to dialysis induction; reasons for dialysis induction, such as fluid overflow and hyperkalemia; and with or without induction of hemodialysis using a central venous catheter.

For cases for which data were not available on the start date of observation, such as urine protein measurement by urine storage practices, data within 3 months after the start date of observation were collected.

The date of observation start was defined as the date of admission for the inpatient group, and the date of the first visit for the non-inpatient group. In the inpatient group, the time from the first visit date to the admission date was measured.

eGFR was calculated using the formula for GFR estimated from serum creatinine in Japan [9].

The mean rate of deterioration of renal function was calculated using the following formula:

Mean rate of deterioration of renal function [mL/min/1.73 m^2^/year] = (eGFR at start of observation − eGFR at start of dialysis) [mL/min/1.73 m^2^]/time from start of observation to start of dialysis [years].

### Statistical analysis

The baseline characteristics are presented as mean ± standard deviation, and the study results are presented as the mean ± standard error. Comparisons of continuous variables were made using *t-*tests with individual variances, and comparisons of nominal variables were made using Fisher’s exact test. A survival analysis was performed to compare the observation period between the inpatient and non-inpatient groups. A log-rank test was performed to determine significant differences. Multivariate analysis was conducted to search for factors significantly associated with the induction of hemodialysis using a central venous catheter. To account for multicollinearity, odds ratios (ORs) for catheter induction of dialysis were calculated by adjusting for eight items: two basic attributes, four background factors at the start of the observation, induction of dialysis due to fluid overflow or hyperkalemia, and history of EH. A two-tailed *P* value of < 0.05 was considered significant. All analyses were performed using JMP9.0.3 (SAS Institute Inc., Cary, NC, USA).

## Results

### Baseline characteristics

Characteristics at the start of observation are shown in Table 1. The mean age was 66.1 ± 13.6 years, 64.3% were male, and the prevalence of diabetes was 43.9%. There were 120 patients in the inpatient group and 51 patients in the non-inpatient group.

**Table 1:** Patient characteristics

	Total		Inpatient group		Non-inpatient group		*P* value
Period from first visit to admission (months)	NA	NA	3.43 ± 9.31	120	NA	NA	NA
Age at starting observation (years old)	66.1 ± 13.6	171	67.1 ± 11.1	120	63.8 ± 18.1	51	0.230
Male (%)	64.3	171	65.8	120	60.8	51	0.601
Diabetes (%)	43.9	171	49.2	120	31.4	51	0.043
Systolic blood pressure (mmHg)	140.6 ± 21.4	170	138.4 ± 19.2	120	145.9 ± 25.4	50	0.032
Diastolic blood pressure (mmHg)	76.6 ± 16.4	168	75.7 ± 15.3	120	78.9 ± 18.7	48	0.302
Hemoglobin (g/dL)	10.66 ± 1.97	171	10.72 ± 2.09	120	10.51 ± 1.66	51	0.490
Uremic acid	7.42 ± 1.94	164	7.33 ± 1.85	117	7.65 ± 2.15	47	0.371
Low-density lipoprotein	104.2 ± 31.6	165	103.0 ± 31.3	118	107.2 ± 32.4	47	0.450
Proteinuria (g/day)	2.43 ± 2.86	170	2.41 ± 3.04	119	2.49 ± 2.42	51	0.848
eGFR at starting observation (mL/min/1.73 m^2^)	21.23 ± 11.57	171	21.42 ± 10.43	120	20.79 ± 14.01	51	0.773
eGFR at starting dialysis (mL/min/1.73 m^2^)	6.99 ± 4.80	171	6.50 ± 4.19	120	8.14 ± 5.86	51	0.075

Mean ± SD, number of patients

**Fig. 2:**
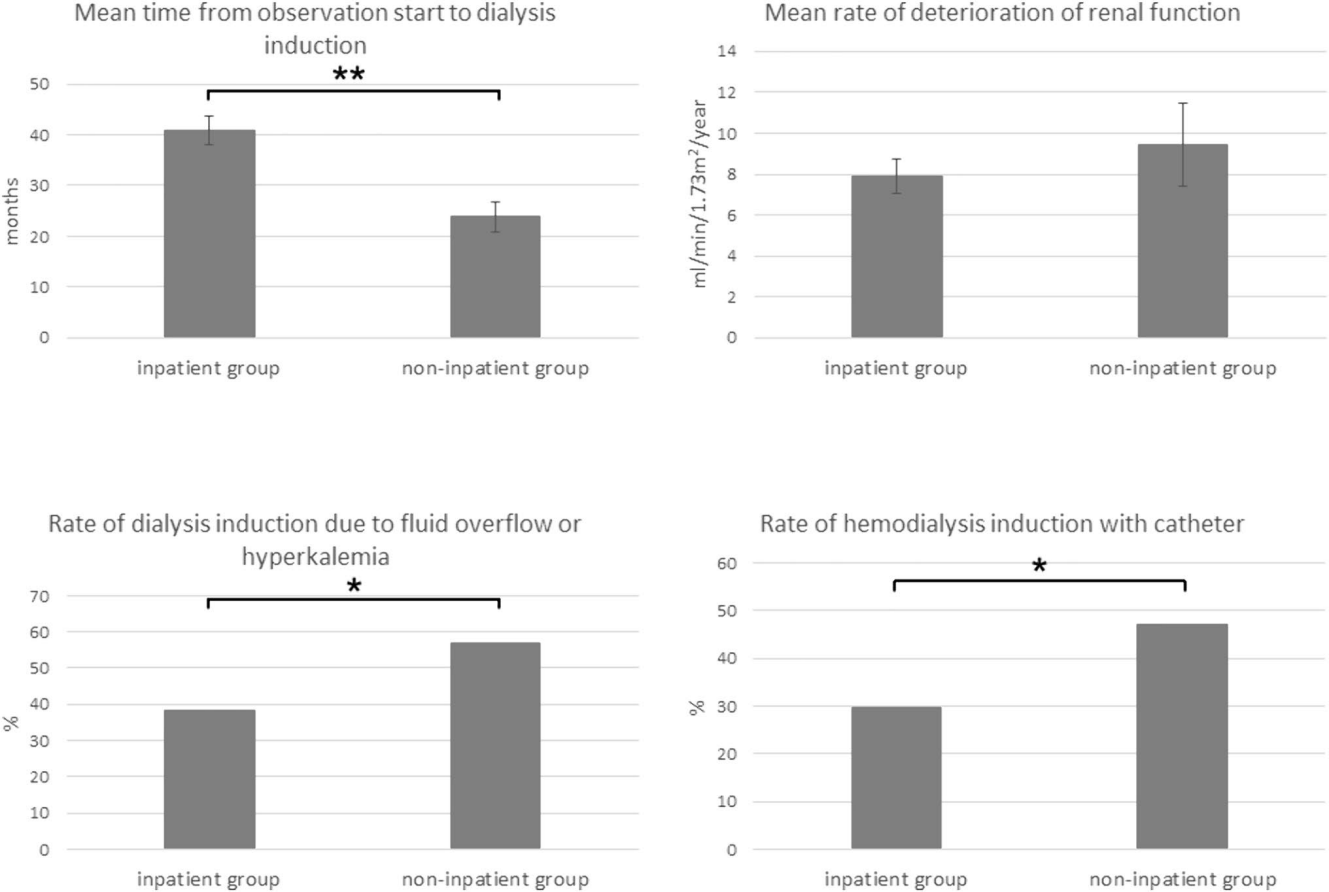
Comparison of the inpatient group and non-inpatient group at the time of dialysis induction. The results represent the mean ± standard error. **P < 0.001 inpatient group vs. non-inpatient group. *P < 0.05 inpatient group vs. non-inpatient group

Figure 2 shows the results of the analysis. The mean time from the start of observation to dialysis induction was (inpatient group vs. non-inpatient group) 40.8 ± 2.8 vs. 23.9 ± 3.0 months (*P* < 0.001); the rate of fluid overflow or hyperkalemia was 38.3 vs. 56.9% (*P* = 0.029); and the rate of induction of hemodialysis using a central venous catheter was 22.5 vs. 47.1% (*P* = 0.002). There was no significant difference in the mean rate of deterioration of renal function (7.9 ± 0.84 vs. 9.5 ± 2.0 mL/min/1.73 m^2^/year, *P* = 0.48).

**Fig. 3:**
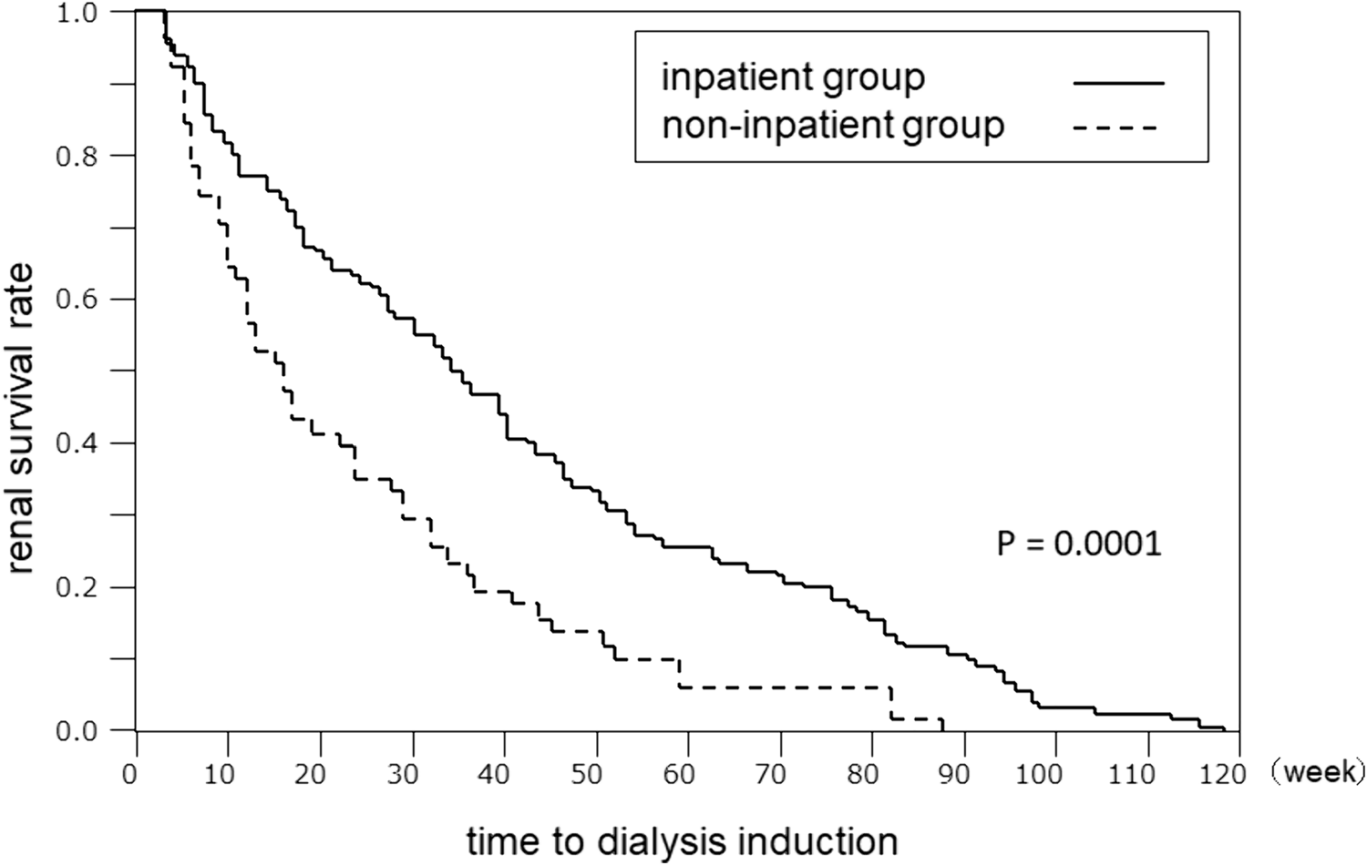
Survival analysis comparing the time from the start of observation to the introduction of dialysis between the inpatient and non-inpatient groups

Figure 3 shows the results of a survival analysis comparing the time from the start of observation to the introduction of dialysis between the two groups. The inpatient group had a significantly longer time from the start of observation to the start of dialysis (*P* < 0.0003).

The result of the multivariate analysis is shown in Table 2. Dialysis induction due to fluid overflow or hyperkalemia was positively associated with hemodialysis induction with a central venous catheter (adjusted OR of 5.36, 95% CI 2.35–12.90, *P* < 0.001). History of EH was inversely associated with hemodialysis induction with central venous catheter (adjusted OR of 0.30, 95% CI 0.13–0.67, *P* = 0.003).

**Table 2:** Factors associated with the induction of hemodialysis using a central venous catheter (multivariate analysis)

	Odds ratio	95% CI	*P* value
Age at start of observation (per 1 year)	1.026	0.993–1.062	0.132
Male	0.602	0.253–1.415	0.246
Systolic blood pressure (per 1 mmHg)	0.988	0.968–1.007	0.224
Hemoglobin (per 1 g/dL)	1.008	0.842–1.255	0.942
Proteinuria (per 1 g/day)	0.851	0.686–1.020	0.116
eGFR at start of observation (per 1 mL/min/1.73m^2^)	0.997	0.963–1.032	0.872
Dialysis induction due to fluid overflow or hyperkalemia	5.364	2.353–12.903	< 0.0001
Having received educational hospitalization	0.298	0.130–0.665	0.004

Dialysis induction due to fluid overflow or hyperkalemia and previous EH were associated with the induction of hemodialysis using a central venous catheter (adjusted OR of 5.36 in dialysis induction due to fluid overflow and hyperkalemia, 95%CI 2.35–12.90, *P* < 0.001; and 0.30 in patients with previous EH, 95%CI 0.13–0.67, *P* = 0.003).

## Discussion

To examine the effect of EH prior to outpatient collaborative care, the present study examined factors associated with dialysis induction in CKD patients starting dialysis. The mean time from the first visit to EH was approximately 3.4 months. Although the observation period was shorter in the inpatient group, we found that the time from the start of observation to dialysis induction in the inpatient group was significantly longer (about 1.7 times) than that in the non-inpatient group. Moreover, the rate of induction of hemodialysis using a central venous catheter was also significantly lower in the inpatient group. Multivariate analysis revealed that risk factors for induction of hemodialysis using a central venous catheter were dialysis induction due to fluid overflow or hyperkalemia, and absence of previous EH.

Multidisciplinary care for CKD has been reported to slow the rate of renal function decline [8]; however, in this study, there was no significant difference in the rate of deterioration of renal function between the two groups. Previous studies that compared the rate of deterioration of renal function before and after an intervention consisting of one-week inpatient multidisciplinary care reported that the rate was lower after the intervention than before it [2, 7]. A previous study also reported that multidisciplinary inpatient care significantly prolonged the duration of stage G5 [6]. However, a study reported that inpatient multidisciplinary care decreased the inhibitory effect on the rate of renal function deterioration over time after discharge from the hospital [7]. This suggests that short-term inpatient intervention alone may have a limited impact on the rate of renal function deterioration. In the present study, the duration of EH in the inpatient group was as short as 1 week. Subsequently, during the observation period, both groups received long-term outpatient multidisciplinary care, which included nurses and dietitians as part of our physician-family physician collaborative care. This may have contributed to the lack of a significant difference in the rate of renal function deterioration between the two groups during the observation period. However, the inpatient group showed a significant reduction in the need for dialysis induction due to fluid overload and hyperkalemia. This suggests that the addition of EH to collaborative care prevented the need for dialysis induction due to fluid overload and hyperkalemia. Furthermore, hyperkalemia and fluid overload were identified as independent factors for unplanned dialysis induction in the multivariate analysis. Dialysis induction due to these factors may accelerate the progression to dialysis because they often necessitate dialysis induction despite residual renal function. Overall, our findings suggest that the combination of EH and outpatient care is more effective than outpatient multidisciplinary care alone in prolonging the time to dialysis.

It has been reported that multidisciplinary care for CKD patients improves life expectancy after dialysis and that planned induction of dialysis is an independent factor that improves life expectancy after dialysis [10]. On the other hand, it has been reported that hemodialysis induction using a central venous catheter decreases life expectancy after dialysis [1].

At our EH, nurses and clinical engineers familiar with dialysis therapy introduce patients to hemodialysis and peritoneal dialysis treatments and show them how dialysis is performed. In the present study, the rate of hemodialysis induction using a central venous catheter was significantly lower in the inpatient group. Thus, patients’ disease knowledge may have been improved by the lecture from our medical staff. The lecture may improve their knowledge of their disease and the importance of having vascular access for dialysis in advance. This may have led to the patients requesting planned dialysis induction. Our findings suggest that patient education by a multidisciplinary team effectively increases the rate of patients requesting vascular access for dialysis in advance, which supports previous research.

There are several limitations associated with the present study. First, there may have been some selection bias in case selection. Patients who received EH likely received more aggressive treatment for their disease and may have responded better to interventions, potentially leading to the planned induction of dialysis. However, coordinating cases was challenging as the study was conducted in parallel with actual clinical practice. Second, EH involves a cost of approximately 80,000 yen (for patients who pay 30% of medical costs) and a week of behavioral restraint. These are considered to be major issues when patients choose whether or not to receive EH. However, this study did not examine the patients’ economic status or occupation; thus, we were unable to eliminate selection bias among patients who decided to receive EH. Third, this study was a retrospective cohort study of patients with CKD in whom dialysis was induced. Therefore, it was not possible to determine whether the results of the study apply to all patients with CKD, including those in whom dialysis was not induced. Fourth, our findings suggest that the improvement of renal prognosis and timely preparation of blood access were largely due to changes in patients' behaviors and attitudes by disease education; however, because we were not able to quantitatively evaluate the behavioral changes in patients as a result of disease education in this study, it was difficult to determine whether the behavioral change caused by the multidisciplinary care contributed to renal prognosis and timely preparation of blood access. Further studies are needed to evaluate behavioral changes in CKD patients by multidisciplinary care. Fifth, the study was conducted on a small number of cases at a single hospital; thus, the results cannot be generalized to all CKD patients.

## Conclusion

One-week inpatient multidisciplinary care for CKD (EH) prior to outpatient collaborative care was effective for postponing dialysis induction and avoiding the induction of hemodialysis using a central venous catheter, a factor that worsens life expectancy in dialysis patients.

### Supplementary Information

Below is the link to the electronic supplementary material.

Supplementary file1 (TIF 123 KB)

Supplementary file2 (DOCX 21 KB)

## Data Availability

The data that support the findings of this study are available from the corresponding author, [Hiroshi Kado], upon reasonable request.
