# Supplementary figures and images for: Effects of 1-week inpatient multidisciplinary care for chronic kidney disease prior to outpatient collaborative care
Source: Clin Exp Nephrol. 2024 Apr 20;28(9):910–6. doi: 10.1007/s10157-024-02496-5 (PMC11341574; doi:10.1007/s10157-024-02496-5)

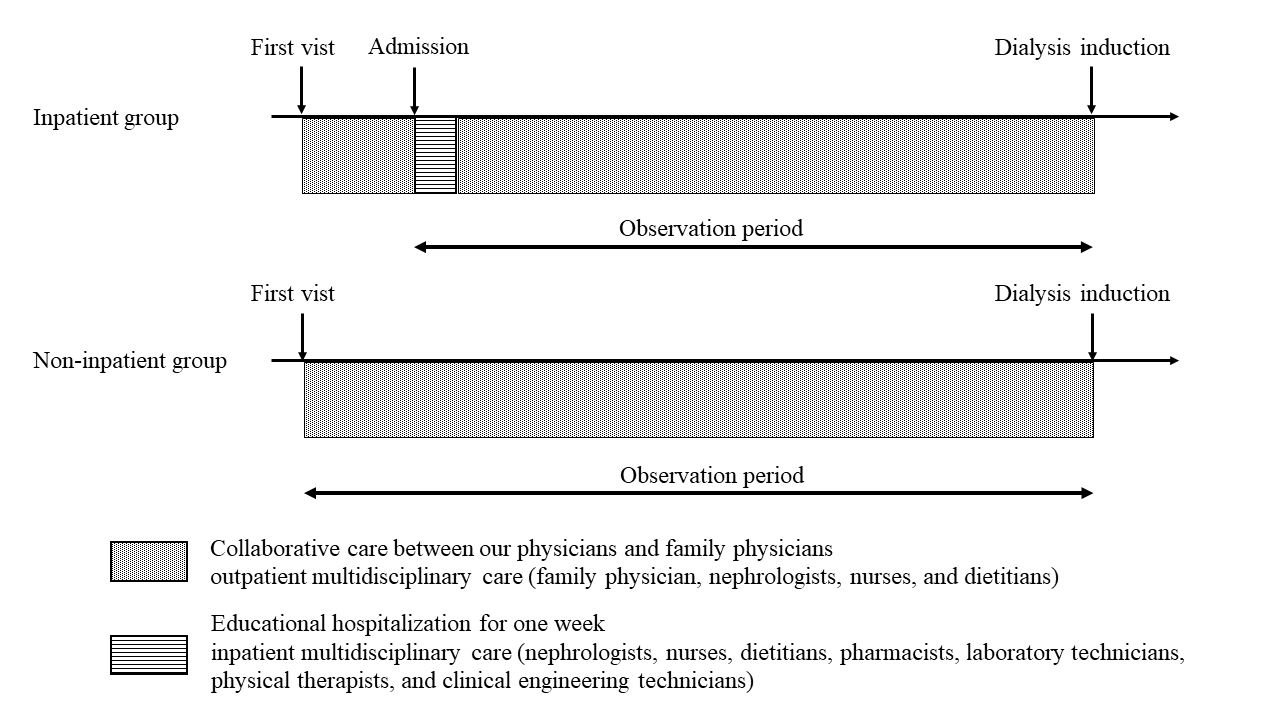

Supplement: Supplementary file 1 — Supplementary file1 (TIF 123 KB) [file 10157_2024_2496_MOESM1_ESM.tif]
