# Supplementary material for: Effects of 1-week inpatient multidisciplinary care for chronic kidney disease prior to outpatient collaborative care
Source: Clin Exp Nephrol. 2024 Apr 20;28(9):910–6. doi: 10.1007/s10157-024-02496-5 (PMC11341574; doi:10.1007/s10157-024-02496-5)
Supplement: Supplementary file 2 — Supplementary file2 (DOCX 21 KB) [file 10157_2024_2496_MOESM2_ESM.docx]

Supplementary Table 1. One-week educational hospitalization schedule in our hospital

|  | | Wed | Thurs | | Fri | | Sat | | Sun | | | Mon | Tue |
| --- | --- | --- | --- | --- | --- | --- | --- | --- | --- | --- | --- | --- | --- |
| Meal | | Salt-restricted diet (5g/day) | | | | | | | | | | | |
| Examination | | Chest Xp  ECG  24-hour Holter ECG  Simple test for sleep apnea syndrome  InBody | | Blood test  Echocardiography | | Renal artery ultrasound  Carotid ultrasonography | |  | |  | Blood test  Pulse wave propagation velocity | |  |
| Lecture | Nephrologist |  | | Pathophysiology and treatment of chronic kidney disease and therapeutic effects of salt reduction | |  | |  | |  |  | | Explanation of test results during hospitalization |
|  | Pharmacist |  | |  | | Drug guidance | |  | |  |  | |  |
|  | Dietitian |  | | Group nutrition counseling | |  | |  | |  | Individual nutritional guidance | |  |
|  | Nurse | Orientation  Correct blood pressure measurement  Taste test | | Description of peritoneal dialysis | | Daily living precautions | |  | |  | Free discussion (patients and nurse) | | Comprehension Check Test |
|  | Laboratory technician | Explanation of laboratory values in blood and urine tests related to CKD | |  | |  | |  | |  |  | |  |
|  | Clinical engineer |  | | Introduction to renal replacement therapy (tour of hemodialysis unit) | |  | |  | |  |  | |  |
|  | Physical therapist |  | |  | | Exercise therapy | |  | |  |  | |  |
| Do it yourself | | Blood pressure measurement (upon waking and before sleep)  Weight measurement (upon waking)  24-hour urine storage | | | | | | | | | | | |
| Video Content | | About chronic kidney disease | | Treatment of chronic kidney disease | | The function of the kidneys | |  | |  | About high blood pressure | |  |
